# *De novo *sequencing and characterization of floral transcriptome in two species of buckwheat (*Fagopyrum*)

**DOI:** 10.1186/1471-2164-12-30

**Published:** 2011-01-13

**Authors:** Maria D Logacheva, Artem S Kasianov, Dmitriy V Vinogradov, Tagir H Samigullin, Mikhail S Gelfand, Vsevolod J Makeev, Aleksey A Penin

**Affiliations:** 1 Department of Evolutionary Biochemistry, A.N. Belozersky Institute of Physico-Chemical Biology, M.V. Lomonosov Moscow State University, Moscow, Russia; 2 Evolutionary Genomics Laboratory, Faculty of Bioengineering and Bioinformatics, M.V. Lomonosov Moscow State University, Moscow, Russia; 3 V.A. Engelhardt Institute of Molecular Biology, Russian Academy of Sciences, Moscow, Russia; 4 A.A. Kharkevich Institute for Information Transmission Problems, Russian Academy of Science, Moscow, Russia; 5 Faculty of Bioengineering and Bioinformatics, M.V. Lomonosov Moscow State University, Moscow, Russia; 6 N.I Vavilov Institute of General Genetics, Russian Academy of Sciences, Moscow, Russia; 7 State Scientific Institute of Genetics and Selection of Industrial Microorganisms, GosNIIgenetika, Moscow, Russia; 8 Department of Genetics, Biological faculty, M.V. Lomonosov Moscow State University, Moscow, Russia

## Abstract

**Background:**

Transcriptome sequencing data has become an integral component of modern genetics, genomics and evolutionary biology. However, despite advances in the technologies of DNA sequencing, such data are lacking for many groups of living organisms, in particular, many plant taxa. We present here the results of transcriptome sequencing for two closely related plant species. These species, *Fagopyrum esculentum *and *F. tataricum*, belong to the order Caryophyllales - a large group of flowering plants with uncertain evolutionary relationships. *F. esculentum *(common buckwheat) is also an important food crop. Despite these practical and evolutionary considerations *Fagopyrum *species have not been the subject of large-scale sequencing projects.

**Results:**

Normalized cDNA corresponding to genes expressed in flowers and inflorescences of *F. esculentum *and *F. tataricum *was sequenced using the 454 pyrosequencing technology. This resulted in 267 (for *F. esculentum*) and 229 (*F. tataricum*) thousands of reads with average length of 341-349 nucleotides. *De novo *assembly of the reads produced about 25 thousands of contigs for each species, with 7.5-8.2× coverage. Comparative analysis of two transcriptomes demonstrated their overall similarity but also revealed genes that are presumably differentially expressed. Among them are retrotransposon genes and genes involved in sugar biosynthesis and metabolism. Thirteen single-copy genes were used for phylogenetic analysis; the resulting trees are largely consistent with those inferred from multigenic plastid datasets. The sister relationships of the Caryophyllales and asterids now gained high support from nuclear gene sequences.

**Conclusions:**

454 transcriptome sequencing and *de novo *assembly was performed for two congeneric flowering plant species, *F. esculentum *and *F. tataricum*. As a result, a large set of cDNA sequences that represent orthologs of known plant genes as well as potential new genes was generated.

## Background

Transcriptome sequencing is a convenient way to rapidly obtain information on the expressed fraction of genome. With the advent of next-generation sequencing transcriptomic data for many species became available. However many important taxonomic groups of living organisms remain underrepresented. This is especially pressing problem for plants since few of them have been a priority for large-scale sequencing projects. The present study aimed at filling the gap in the genomic data for the genus *Fagopyrum*, a group of plants important for both practical and theoretical reasons.

*Fagopyrum *(buckwheat) belongs to the eudicot family Polygonaceae. The genus *Fagopyrum *comprises about 17 species; one of the species, *F. esculentum *(common buckwheat), is a crop and honey-producing plant widely cultivated in several countries, in particular, Canada, China, Japan, Russia and Ukraine. Recently, an extensive search for the wild ancestor of the cultivated buckwheat identified the ancestral group, called *F. esculentum *subspecies *ancestrale *[1,2]. Two more distant relatives of the cultivated buckwheat are *F. homotropicum *and *F. tataricum *[3]. Both of them have an important favorable trait absent in the common buckwheat, the ability to self-pollination (self-compatibility). Thus they have a potential for use in breeding for the development of self-compatible cultivars.

Despite its economic importance, molecular studies on buckwheat are few. They are mainly confined to the development of molecular markers based on anonymous DNA sequences (RAPD, SSR, AFLP) and the characterization of several proteins [4-6]. Also, the molecular systematics of the genus *Fagopyrum *was extensively studied using nuclear and plastid sequences [7-9], and a complete sequence of the chloroplast genome of the common buckwheat was reported recently [10]. These data, however, have only limited applicability for the buckwheat breeding and improvement. The identification of genes expressed during flower development can enable the search for candidate genes responsible for agriculturally important traits.

The utility of large-scale buckwheat gene sequencing data is not limited to their potential practical applications. They will also contribute to the plant comparative and evolutionary genomics. Not only the buckwheat, but the entire family Polygonaceae was out of scope of molecular genetic studies. The Polygonaceae is a middle-sized family (*ca*. 1000 species) characterized by several morphological features that are not typical for other eudicots. Among them is an unusual floral structure with flowers having a uniseriate perianth not differentiated into sepals and petals. This feature, as well as the trimerous perianth found in several Polygonaceae genera and considered as ancestral state for the family, is common for monocots and basal angiosperms but rarely found in eudicots.

The genetic control of the floral development has been well studied in several model plants, primarily *Arabidopsis thaliana *and some gene interactions have been found to be conserved in a variety of species [11]. However, it is widely accepted that for understanding of the plant morphological evolution new model systems representing different lineages of the angiosperms should be selected [12,13]. Given the morphological peculiarities of Polygonaceae, *Fagopyrum*, and specifically, *F. esculentum*, a fast growing annual weed with high seed production, is a plausible candidate for such a system.

**Figure 1:**
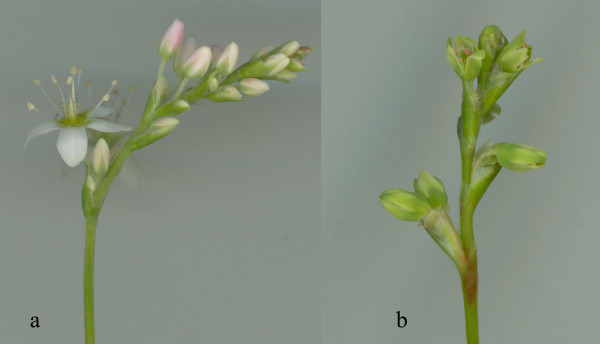
**Fragment of inflorescence of *Fagopyrum esculentum *ssp**. *ancestrale *(a) and *F. tataricum *(b).

Many factors are important for species to be useful as a model system, a crucial one being the availability of genomic and transcriptomic data that enable efficient analysis of gene structure, expression and evolution. Until recently, such data were available only for major model species such as *A. thaliana *and rice, and for several species of basal angiosperms [14]. We report characterization of genes expressed in the flower and inflorescence of two species of buckwheat, the common buckwheat *F. esculentum *and *F. tataricum*. This choice was based on the reasons outlined above. One is the economical importance of *F. esculentum*; and *F. tataricum *being a potential donor of agriculturally important traits such as self-compatibility and resistance to environmental stresses [15] to the common buckwheat. The other stems from the evolutionary developmental genetics (evo-devo) considerations. These two species have contrasting floral morphology: *F. esculentum *has petaloid tepals and *F. tataricum *has sepaloid ones (Figure 1). Further qualitative and quantitative analysis based on the transcriptome data may help to reveal the mechanisms that are responsible for the morphological difference between these species.

We expect that characterization of buckwheat transcriptomes will contribute not only to the genetics of this genus. The order Caryophyllales is characterized by a striking diversity of the floral structure and the perianth evolution that can hardly be explained based only on the knowledge about classic model species [16]. Few transcriptome datasets of the Caryophylalles are available to date [17,18]. Publicly available, annotated set of buckwheat genes expressed in the flower and inflorescence resulting from this study will facilitate comparative and evolutionary studies within the Caryophylalles, important for the plant evo-devo field.

## Results

### 454 sequencing and assembly

Pyrosequencing of normalized cDNA libraries resulted in 266782 reads for *F. esculentum *and 229031 for *F. tataricum *with the average length of 349 and 341 nucleotides respectively. The raw data were deposited in the NCBI Sequence Read Archive (SRA) under the accession number SRA023408.

**Figure 2:**
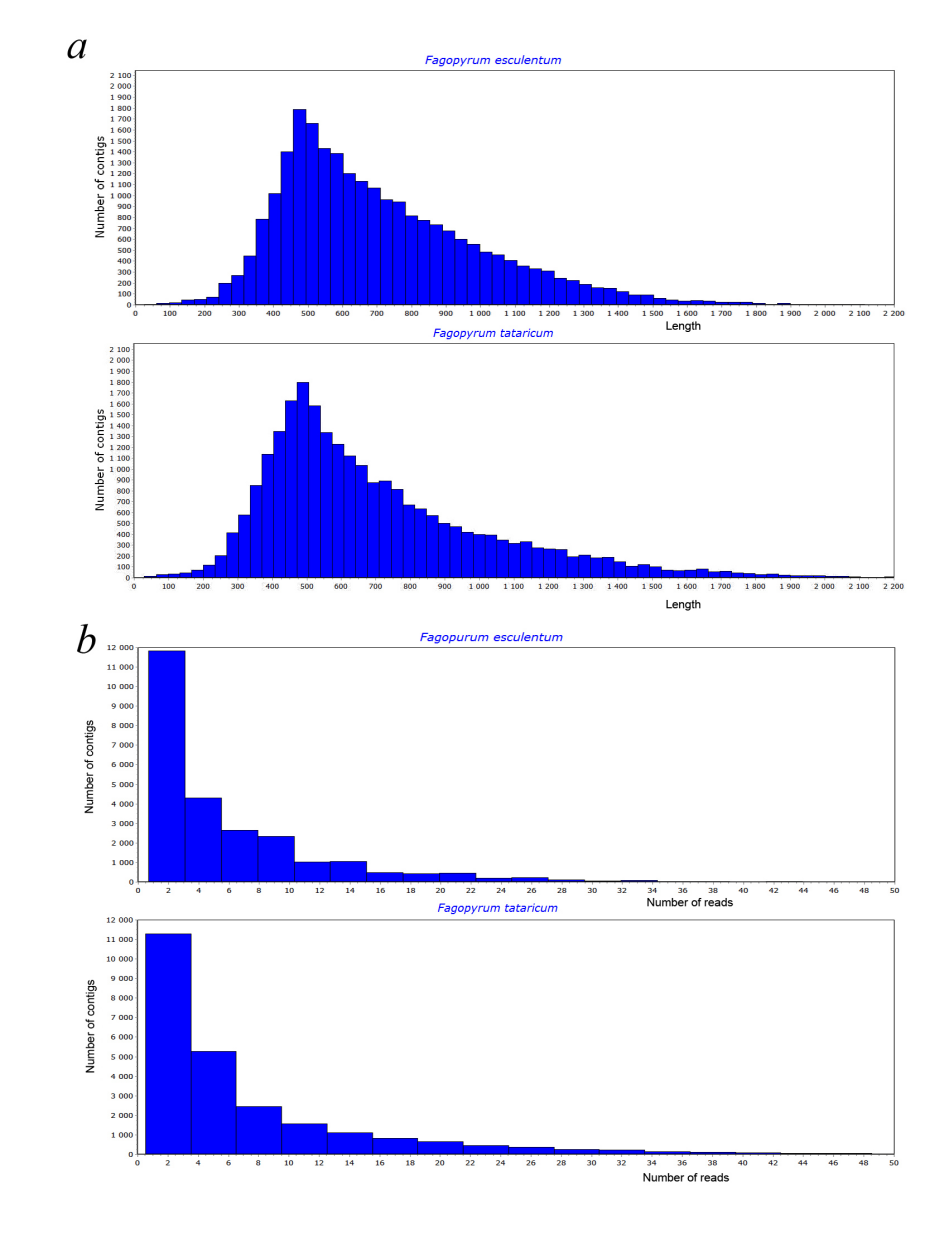
***De novo *sequencing and assembly characteristics**. Distribution of contig lengths (a), distribution of the number of reads per contig (b).

After assembly, 25435 and 25401 contigs were obtained for *F. esculentum *and *F. tataricum*, respectively, while 56874 and 42913 reads were retained as singletons (the sequences of contigs and singletons are available in the Additional files 1 and 2). The average contig length and other characteristics of the assembly are given in Table 1 (see also Figure 2). The sequencing coverage (estimated as the mean number of reads per contig) was assessed as 8.2 for *F. esculentum *and 7.5 for *F. tataricum *(Figure 2).

**Table 1:** Characteristics of raw data and contigs

	Fagopyrum esculentum	Fagopyrum tataricum
Number of reads	266782	229031

Length, mean (min-max)	349 (40-971)	340 (40-976)

Number of contigs	25435	25401

Length, mean (min-max)	698.4 (42-3607)	703 (46-3298)

Number of reads per contig, mean (min-max)	8.2 (2-224)	7.5 (2-295)

Since several full-length coding sequences of buckwheat genes were available in GenBank, we used this information to assess the assembly quality by mapping the contigs onto these sequences. Of 34 available sequences of *F. esculentum*, 30 were found in our assembly. In the latter, the average fraction of coding nucleotides covered by at least one read was 73.4%; while for 11 genes the coding sequence was covered completely (Additional file 3). This demonstrates that the assembled contigs can be a useful source of complete or near-complete coding sequences for *F. esculentum*.

### Annotation

Contig sequences were compared to the non-redundant protein database and about two thirds of them had significant matches (see Table 2 for exact values). In both *F. esculentum *and *F. tataricum*, the species that provided most of the top BLAST hits was *Vitis vinifera *(about 7 thousands of *Fagopyrum *genes had the strongest similarity to *V. vinifera *genes). The *Vitis *nuclear genome has been recently sequenced [19]. The next closest species were another plant with completely sequenced nuclear genome, *Populus trichocarpa *[20], and *Ricinus communis*, whose genome sequencing project is now in progress [21]. A similar taxonomic distribution of BLAST hits has been found in a transcriptome of an asterid, *Crataerostigma plantagineum *[22].

**Table 2:** Summary of BLAST search and annotation

	***Fagopyrum esculentum***	***Fagopyrum tataricum***
**Contigs**		

Number of contigs with BLAST match	19122	19072

Number of contigs with GO annotation	15115	14684

Number of contigs without BLAST matches	6313	6329

Number of contigs with predicted ORF/mean length of ORF, bp	25345/368 (30-1637) 25115 > 90	24984/390 (39-1983) 24743 > 90

Species provided most of all best BLAST hits	Vitis vinifera	Vitis vinifera

**Singletons**		

Total number of singletons	56766	42899

Number of singletons with BLAST match	14260	14671

Number of singletons with GO annotation	10303	10448

Number of singletons without BLAST matches	42506	28228

Species provided most of all best BLAST hits	Vitis vinifera	Vitis vinifera

For the singletons, the fraction of sequences that had BLAST matches was lower than for the contigs (Table 2), as expected given their smaller length [23]. The taxonomic distribution of species that provided most top hits was the same as for the contigs.

An automatic ORF prediction revealed open reading frames with length more than 90 bp in about 98% contigs in both buckwheat species. This length, though being much less than the average length of protein-coding sequence in plants, is higher than expected to arise by chance (assessed by random shuffling of the contigs with subsequent ORF prediction) suggesting that most of the contigs correspond to protein-coding genes. This is consistent with the method used for cDNA synthesis, as it is based on the presence of polyA-tail on the 3'-end of most mRNAs, the library is highly enriched in mRNA sequences. Interestingly, most contigs that had no BLAST matches, also contained predicted ORFs with length exceeding the one expected for a random sequence of a similar GC-content (data not shown).

**Figure 3:**
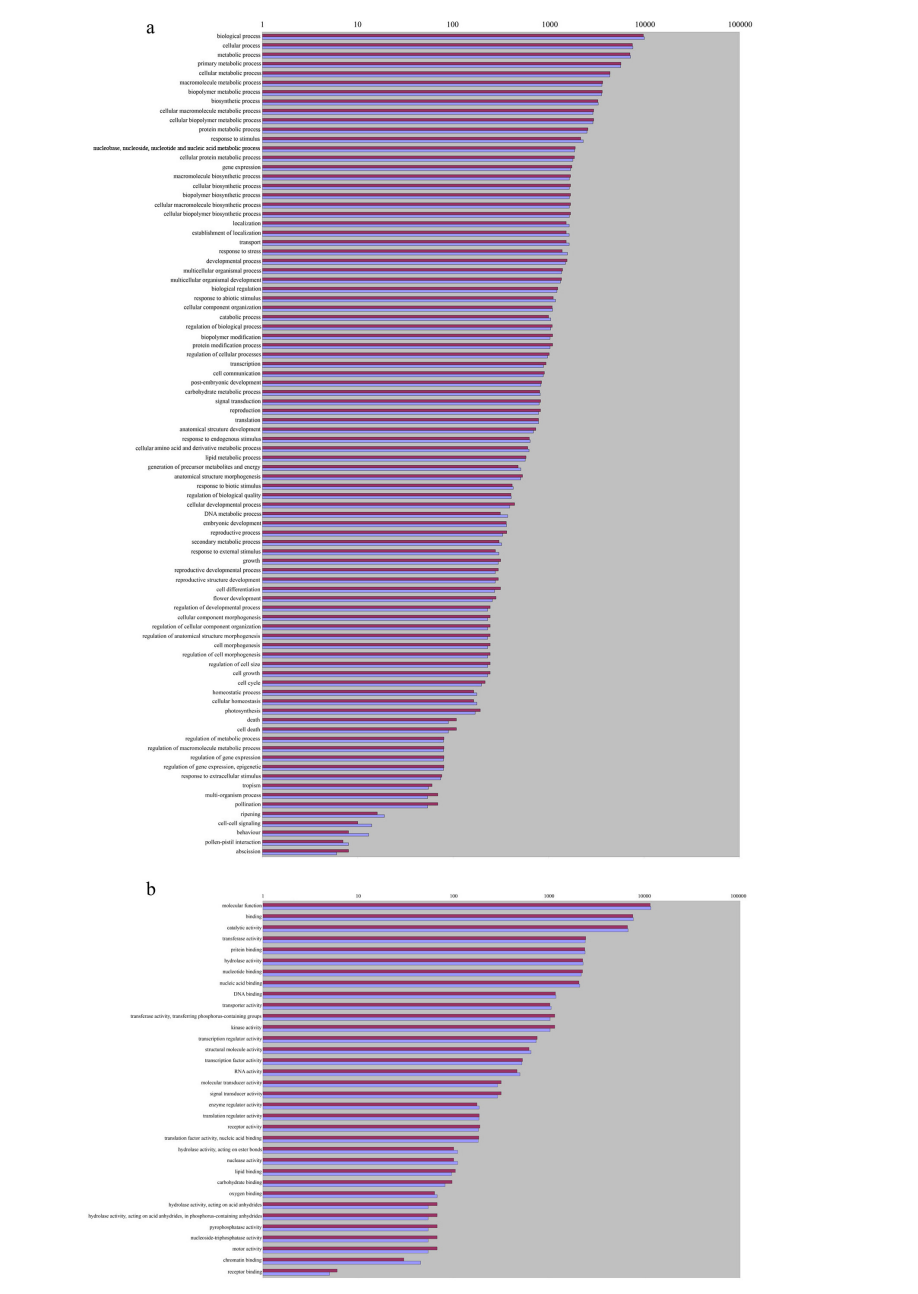
**Distribution of Gene Ontology categories for Biological Process (a) and Molecular Function subontologies (b) for *F. esculentum *(violet bars) and *F. tataricum *(blue bars)**.

To obtain a functional annotation of the buckwheat genes, we used Gene Ontology (GO, [24]). For both species of buckwheat, about 60% contigs were annotated. This fraction is similar to or exceeds the annotated fraction in other recently published 454-derived transcriptomes, for example those of pine [23] and ginseng [25]. The distribution of GO categories is very similar in *F. esculentum *and *F. tataricum*, with no categories showing significant differences between the species (Figure 3).

The contigs that had no BLASTX hits but contained an ORF with a length exceeding 90 nucleotides were searched against the Pfam database (e-value threshold 1). A similar number of translated contigs had significant match in the database for both species, 1795 for *F. esculentum *and 1775 for *F. tataricum*, with 1108 and 1156, respectively, having a match in pfamA families. Among the latter, about 50% (47% for *F. esculentum *and 50% for *F. tataricum*) had hits to families containing at least one protein from plants.

Since one of our aims was to identify genes that are responsible for the flower development, we specifically searched for buckwheat orthologs of genes and gene families that are known to be involved in the flower development in *Arabidopsis*. Candidate orthologs were identified as bidirectional best BLAST hits. Such orthologs were found (in either *F. esculentum *or *F. tataricum*) for 61 out of 141 genes of *Arabidopsis*. Among them were all genes controlling the floral organ identity (ABC model genes) and some other MADS-box genes; genes controlling stem cell maintenance (*CLAVATA1*, *CLAVATA2 *and *WUSCHEL*); negative regulators of *AGAMOUS *(*APETALA2*, *LEUNIG*, *SEUSS*, *BELLRINGER*); genes of the *YABBY *family; auxin response factors and some others (Table 3).

**Table 3:** Putative orthologs of buckwheat genes involved in flower development (defined according to Alvarez-Buylla et al. 2008) identified by bidirectional BLAST search

Arabidopsis gene	number	F. esculentum	F. tataricum
APETALA1	AT1G69120	Fagopyrum_esculentum_MIRA_VER3_c1474	Fagopyrum_tataricum_MIRA_VER3_c880

APETALA2	AT4G36920	Fagopyrum_esculentum_MIRA_VER3_c15879	Fagopyrum_tataricum_MIRA_VER3_c21103

APETALA3	AT3G54340	Fagopyrum_esculentum_MIRA_VER3_c5503	Fagopyrum_tataricum_MIRA_VER3_c2664

PISTILLATA	AT5G20240	Fagopyrum_esculentum_MIRA_VER3_c1334	Fagopyrum_tataricum_MIRA_VER3_c2417

AGAMOUS	AT4G18960	Fagopyrum_esculentum_MIRA_VER3_c3409	Fagopyrum_tataricum_MIRA_VER3_c2578

SEPALLATA1	AT5G15800	Fagopyrum_esculentum_MIRA_VER3_c876	Fagopyrum_tataricum_MIRA_VER3_c232

SEPALLATA3	AT1G24260	Fagopyrum_esculentum_MIRA_VER3_c1493	Fagopyrum_tataricum_MIRA_VER3_c2041

WUSCHEL	AT2G17950	Fagopyrum_esculentum_MIRA_VER3_c13614	

SUPERMAN	AT3G23130	Fagopyrum_esculentum_MIRA_VER3_c21331	Fagopyrum_tataricum_MIRA_VER3_c11424

ARGONAUTE1	AT1G48410	Fagopyrum_esculentum_MIRA_VER3 _c1224	Fagopyrum_tataricum_MIRA_VER3_c788,Fagopyrum_tataricum_MIRA_VER3_c5744

TSO1	AT3G22780		Fagopyrum_tataricum_MIRA_VER3_c13853

CLAVATA1	AT1G08590		Fagopyrum_tataricum_MIRA_VER3_c8814

CLAVATA2	AT1G65380	Fagopyrum_esculentum_MIRA_VER3_c20999	Fagopyrum_tataricum_MIRA_VER3_c16742

FASCIATA1	AT1G65470	Fagopyrum_esculentum_MIRA_VER3_c10249	Fagopyrum_tataricum_MIRA_VER3_c18675

FASCIATA2	AT5G64630		Fagopyrum_tataricum_MIRA_VER3_c21840

SHOOT MERISTEMLESS	AT1G62360		Fagopyrum_tataricum_MIRA_VER3_c5954

ULTRAPETALA1	AT4G28190	Fagopyrum_esculentum_MIRA_VER3_c14864	Fagopyrum_tataricum_MIRA_VER3_c12493

WIGGUM/ENHANCED RESPONSE TO ABSCISIC ACID1	AT5G40280	Fagopyrum_esculentum_MIRA_VER3_c7673	Fagopyrum_tataricum_MIRA_VER3_c5603

LEUNIG	AT4G32551	Fagopyrum_esculentum_MIRA_VER3_c11836	Fagopyrum_tataricum_MIRA_VER3_c4980

LEUNIG HOMOLOG	AT2G32700		Fagopyrum_tataricum_MIRA_VER3_c7645

SEUSS	AT1G43850	Fagopyrum_esculentum_MIRA_VER3_c14461	

YABBY1	AT2G45190	Fagopyrum_esculentum_MIRA_VER3_c9839	

AS1	AT2G37630	Fagopyrum_esculentum_MIRA_VER3_c2262	Fagopyrum_tataricum_MIRA_VER3_c326

AUXIN RESPONSE FACTOR 5/MONOPTEROS	AT1G19850	Fagopyrum_esculentum_MIRA_VER3_c12988	

PHABULOSA	AT2G34710		Fagopyrum_tataricum_MIRA_VER3_c6862

YABBY3	AT4G00180		Fagopyrum_tataricum_MIRA_VER3_c6676

AINTEGUMENTA	AT4G37750		Fagopyrum_tataricum_MIRA_VER3_c9095

ARF3/ETTIN	AT2G33860		Fagopyrum_tataricum_MIRA_VER3_c22133

PGP19	AT3G28860		Fagopyrum_tataricum_MIRA_VER3_c2013

PERIANTHIA	AT1G68640	Fagopyrum_esculentum_MIRA_VER3_c7084	

PETAL LOSS	AT5G03680		Fagopyrum_tataricum_MIRA_VER3_c8866

PIN-FORMED1	AT1G73590		Fagopyrum_tataricum_MIRA_VER3_c3716

PIN-FORMED3	AT1G70940	Fagopyrum_esculentum_MIRA_VER3_c10169	Fagopyrum_tataricum_MIRA_VER3_c355

PINOID	AT2G34650		Fagopyrum_tataricum_MIRA_VER3_c4499

TOUSLED	AT5G20930		Fagopyrum_tataricum_MIRA_VER3_c11648

ABORTED MICROSPORES	AT2G16910		Fagopyrum_tataricum_MIRA_VER3_c4925

AUXIN RESPONSE FACTOR 6	AT1G30330	Fagopyrum_esculentum_MIRA_VER3_c14779	Fagopyrum_tataricum_MIRA_VER3_c11412

AUXIN RESPONSE FACTOR 8	AT5G37020	Fagopyrum_esculentum_MIRA_VER3_c6633	Fagopyrum_tataricum_MIRA_VER3_c11597

BARELY ANY MERISTEM 1	AT5G65700	Fagopyrum_esculentum_MIRA_VER3_c2493	Fagopyrum_tataricum_MIRA_VER3_c95

CORONATINE INSENSITIVE 1	AT2G39940	Fagopyrum_esculentum_MIRA_VER3_c11571	Fagopyrum_tataricum_MIRA_VER3_c15577

DELAYED DEHISCENCE1/OPDA-REDUCTASE 3	AT2G06050	Fagopyrum_esculentum_MIRA_VER3_c3772	Fagopyrum_tataricum_MIRA_VER3_c566

FATTY ACID DESATURASE 7	AT3G11170	Fagopyrum_esculentum_MIRA_VER3_c1351	Fagopyrum_tataricum_MIRA_VER3_c705

FAD8	AT5G05580		Fagopyrum_tataricum_MIRA_VER3_c705

GA INSENSITIVE DWARF1A	AT3G05120		Fagopyrum_tataricum_MIRA_VER3_c963

GA INSENSITIVE DWARF1B	AT3G63010	Fagopyrum_esculentum_MIRA_VER3_c7038	

GA INSENSITIVE DWARF1C	AT5G27320		Fagopyrum_tataricum_MIRA_VER3_c963

MALE STERILE1	AT5G22260		Fagopyrum_tataricum_MIRA_VER3_c18359

MYB21	AT3G27810		Fagopyrum_tataricum_MIRA_VER3_c8063

RECEPTOR-LIKE PROTEIN KINASE 2	AT3G02130	Fagopyrum_esculentum_MIRA_VER3_c5605	Fagopyrum_tataricum_MIRA_VER3_c21802

BREVIPEDICELLUS/KNAT1	AT4G08150	Fagopyrum_esculentum_MIRA_VER3_c13223	Fagopyrum_tataricum_MIRA_VER3_c9641

CRABS CLAW	AT1G69180	Fagopyrum_esculentum_MIRA_VER3_c1676	

NGATHA1	AT2G46870		Fagopyrum_tataricum_MIRA_VER3_c8303

NO TRANSMITTING TRACT	AT3G57670	Fagopyrum_esculentum_MIRA_VER3_c16587	Fagopyrum_tataricum_MIRA_VER3_c12724

BLR	AT5G02030		Fagopyrum_tataricum_MIRA_VER3_c2133

STK	AT4G09960		Fagopyrum_tataricum_MIRA_VER3_c6138

DICER-LIKE1	AT1G01040		Fagopyrum_tataricum_MIRA_VER3_c24560

SPATULA	AT4G36930	Fagopyrum_esculentum_MIRA_VER3_c7255	Fagopyrum_tataricum_MIRA_VER3_c22310

STY1	AT3G51060	Fagopyrum_esculentum_MIRA_VER3_c19680	Fagopyrum_tataricum_MIRA_VER3_c6938

STY2	AT4G36260		

BIG BROTHER	AT3G63530	Fagopyrum_esculentum_MIRA_VER3_c12106	Fagopyrum_tataricum_MIRA_VER3_c5454

HWS	AT3G61590	Fagopyrum_esculentum_MIRA_VER3_c12481	

Since this type of search is not able to detect duplications, we performed a manual inspection of the results of BLAST search for several gene families. For the B-class MADS-box gene, *APETALA3 *(*AP3*), we were able to find two highly similar sequences. The similarity between them is lower than that expected for alleles, with multiple indels and non-synonymous substitutions. Thus we assume that they represent paralogs arisen from a recent duplication. In another species from the same family, *Rumex acetosa*, two genes with high similarity to AP3 were also found [26]. Hence, this duplication likely occured in the common ancestor of the Polygonaceae.

### Comparative analysis of the buckwheat transcriptomes

**Figure 4:**
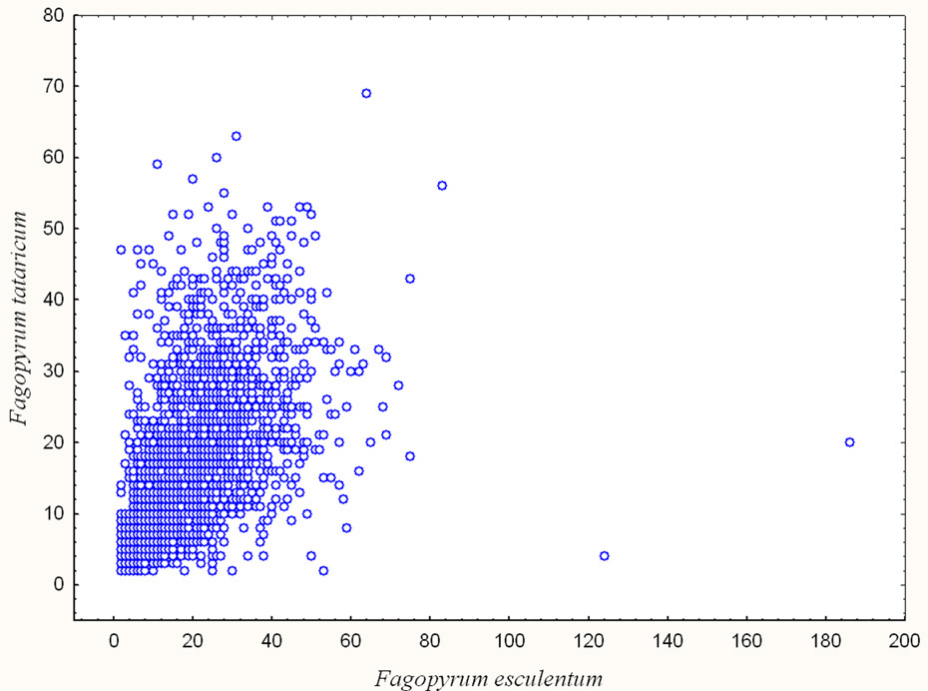
**Number of reads in ortologous contigs (only contigs with length > 300 bp and sequence similarity > 90% are taken into account)**. Each dot represents a pair of orthologs.

The contig assembly and annotation characteristics, as well as the raw data characteristics, are similar for *F. esculentum *and *F. tataricum*. Moreover, the data indicate that there is a strong correlation between the number of reads constituting contigs for orthologous genes of *F. esculentum *and *F. tataricum *(Figure 4). If a contig of a certain gene is overlapping with a certain number of reads, there is a high probability that the contig of its ortholog from the other species is overlapping with a close number of reads.

We addressed the question whether these transcriptomes completely consist of orthologous genes or there are genes expressed in one species but with orthologs that are not represented (absent or not expressed) in the other species. Such transcripts are plausible candidates for the determinants of morphological differences between the species.

To identify these putative differentially expressed genes we first performed a bidirectional BLAST search of the *F. esculentum *and *F. tataricum *contigs against each other. Those contigs that had no match in the other species (either in contigs or in singletons) were regarded as potential differentially expressed genes (PDEGs). Starting bidirectional BLAST search from either species, a similar number of PDEGs was found: 4245 in *F. esculentum *and 4255 in *F. tataricum*. The PDEG fraction of both transcriptomes is enriched in contigs that have no GenBank hits (Table 4) and GO annotations were assigned to only 1132 contigs in *F. esculentum *and 1588 contigs in *F. tataricum*. The distribution of GO categories in both PDEG sets is largely similar, with only few categories being unique for either species (Additional file 4). These categories are represented by a small number of contigs. In most cases the number of contigs representing a category is higher in *F. tataricum *- presumably, because of higher total number of GO-annotated contigs in this species. However, one category, "DNA metabolic process", is represented by a considerably higher number of contigs in *F. esculentum *(79) than *F. tataricum *(49). In both species, the contigs from this category are almost exclusively represented by sequences with high similarity to retrotransposons. Thus there is strong evidence that *F. esculentum *and *F. tataricum *differ in the number or expression of retrotransposons.

**Table 4:** Characteristics of potential differentially expressed genes (PDEG) fraction of buckwheat transcriptomes

	***Fagopyrum esculentum***	***Fagopyrum tataricum***
total number of PDEG	4245	4255

without BLAST matches	2567	2037

with GO annotation	1132	1588

number of contigs covered by more than 10 reads	158	52

In addition to the direct comparison of the PDEG sets, we addressed the question whether they represent random subsets of the overall transcriptomic datasets. To test this, the enrichment analysis was performed, with the PDEG set taken as a test group and the overall set taken as a reference group (p = 0.05). No enrichment by any specific category was found for *F. tataricum*; while several categories were found to be overrepresented in the *F. esculentum *PDEG set (Table 4), in particular categories obviously related to retrotransposons (DNA integration, RNA-directed DNA polymerase activity) and to processes inherent to plants such as sugar metabolism, signaling and amino acid transport.

When we observe that a certain gene in either species is sampled by at least one read but its ortholog in the other species is not sampled this does not necessarily means the absence of expression of this gene in the other species. It might be not sampled due to technical issues related to the processing of RNA and cDNA before sequencing. Taking this into account we performed a statistical assessment of the fraction of such cases (false positives). Since there is a correlation between the number of reads constituting contigs of orthologous genes (see above), the expected number of false positives differs for different levels of coverage. Our analysis indicates that only about 25-30% of PDEG indeed represent genes whose orthologs are not expressed in the other species. For genes that are represented by a high number of reads in one species the probability is much higher (Table 5).

**Table 5:** Expected number of differentially expressed genes in the PDEG sets

***Fagopyrum esculentum***		
**number of reads covering a contig**	**observed number of PDEG**	**expected number of differentially expressed genes**

2	2311	524.672773

3	788	393.71303

4	342	242.460795

5	207	178.845566

6	134	121.243316

7	82	78.2933171

8	61	59.094585

> 8	320	320

*Fagopyrum tataricum*		

2	2456	692.698531

3	838	228.927978

4	406	196.396042

5	183	139.219951

6	110	99.429005

> 6	262	262

### Shared single-copy genes in the 454 transcriptome assemblies and their phylogenetic analysis

**Figure 5:**
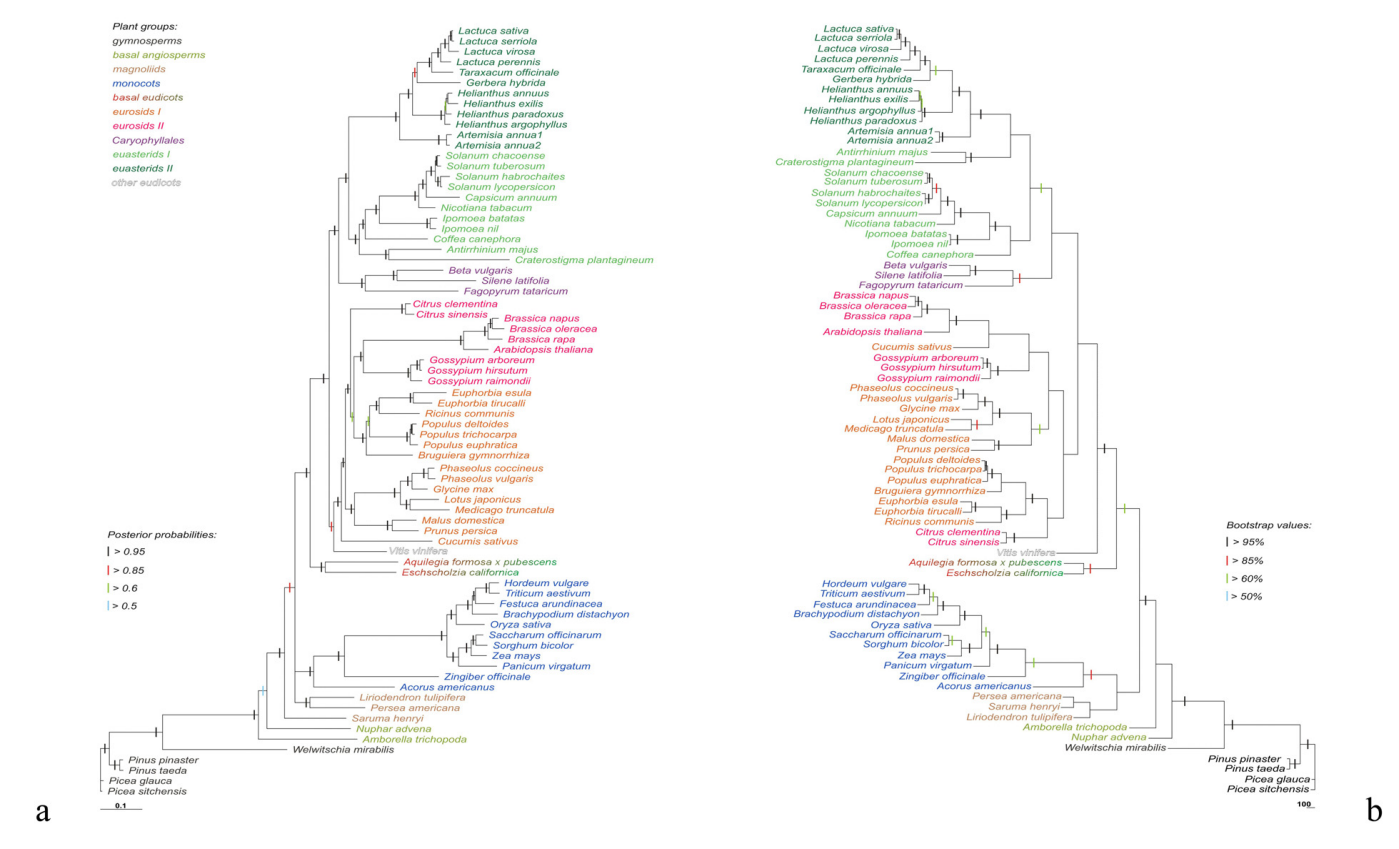
**Trees inferred from the Bayesian analysis (a) and from the maximum parsimony analysis (b) of nucleotide sequences of 13 single-copy genes**. Branch lengths are proportional to the number of expected nucleotide substitutions; scale bar corresponds to one substitution per ten sites for the Bayesian tree and to 100 changes for maximum parsimony tree. Posterior probability and bootstrap values (greater than 0.5 and 50% respectively) are indicated by the colored bars placed on branches.

Large-scale transcriptome data are a potential source of information for multigene phylogenetic analysis (the phylogenomic approach). To adopt its use in plant phylogenetics, Duarte et al. [27] identified a set of single-copy genes shared between *Arabidopsis*, *Populus*, *Vitis *and *Oryza*. Only few of them were well represented in EST assemblies across the major lineages of angiosperms. Though being quite small (13 genes), this subset produced well-resolved tree topologies similar to those inferred in many recent phylogenetic studies. To provide further validation of this approach and to assess the utility of 454 sequencing data for phylogenomics we performed a search for the orthologs of these genes in five 454 transcriptome assemblies. Two of them are those reported in this study - *F. tataricum *and *F. esculentum*, the third one is from another Caryophyllales species - *Silene latifolia *(sequences provided by D. A. Filatov). Taking into account that the analysis of plastid genes indicates on the affinity of Caryophyllales and asterids [10,28] two recently published transcriptomes of asterids: *Craterostigma plantagineum *[22] and *Artemisia annua *[29] were also included in the analyses. For all species almost all genes were present: 13 in *Artemisia annua*, 12 in *F. tataricum*, *Craterostigma plantagineum *and *Silene latifolia *and 11 in *F. esculentum*. For most genes, only one sequence with high similarity to a certain gene was found thus suggesting that they are single-copy in the species sampled. The only exception is *Artemisia annua*, where each of two genes - the orthologs of AT4G08230 and AT5G63135 - is represented by two sequences with high similarity to the *Arabidopsis *gene but differing one from another by multiple nucleotide substitutions and indels. We assume that these sequences represent paralogs emerged from the recent duplication (presumably, duplication occurred at the level of the genus since no paralogs were found in the transcriptomes of another species from Asteraceae). Such «shallow paralogs» do not necessarily adversely affect phylogenetic reconstruction [30] so they were included in the analysis (under the names of *Artemisia annua*1 and *Artemisia annua*2). The sequences were added to the dataset from [27]. The resulting 73-taxon nucleic acid sequences dataset contained 7869 characters (including gaps). The phylogeny was reconstructed using the maximum parsimony (MP) and Bayesian inference (BI) methods based on both nucleotide and amino acid sequences. As an alternative to the combined approach a super distance matrix (SDM) analysis was also applied (only to nucleotide sequences). The trees resulting from MP and BI were similar to those inferred by Duarte and coworkers [27] in topology and resolution (Figure 5). The maximum parsimony trees inferred from both nucleotide and amino acid sequence data were much less resolved, and much less consistent with the current knowledge on the angiosperm phylogeny. The Bayesian trees were well resolved, with posterior probabilities higher than 0.5 for all nodes in the amino acid tree and all but one in the nucleotide tree. In general, the Bayesian tree is similar to those inferred from the analysis of several genes and from the multigene analysis of plastid datasets [28], with *Amborella trichopoda *and *Nuphar advena *being basal among angiosperms, and monocots, eurosids and asterids resolved as monophyletic. Nonetheless there are some points of incongruence, primarily the position of magnoliids and non-monophyly of eurosids I and II.

An interesting result is the position of the Caryophyllales. With the addition of *Fagopyrum *and *Silene*, the sister relationships of the Caryophyllales gained high support (PP = 0.96 for nucleotide data and 1 for amino acid) in the Bayesian analysis. This is consistent with our previous data on the plastid genome phylogeny [10], but in the nuclear gene analysis this topology is present as highly supported for the first time. In the dataset of Duarte et al. [27], where the only caryophyllid species, *Beta vulgaris *was present, it was also grouped with asterids in the maximum likelihood tree but with very a low support (< 50%).

The supertree resulting from SDM is in most points congruent with the Bayesian trees and also supports sister relationships of the Caryophyllales and asterids (Additional file 5).

## Discussion

### Utility of 454 sequencing for gene discovery in non-model species

Despite the economical importance of buckwheat and the great advances in DNA sequencing technologies no genomic data were available to date for any of the species of *Fagopyrum*, with the exception of the complete chloroplast genome sequence of *F. esculentum *ssp. *ancestrale *[10]. Prior to this study, the total number of sequences in GenBank was 149 for *F. esculentum *and 121 (including 83 unannotated EST) for *F. tataricum*. Sequencing and assembly of genes expressed in flower and inflorescence presented in this study results in 25 thousand contigs for each species. Given that the average number of genes encoded in a plant nuclear genome is about 30 thousands (as estimated from seven completely sequenced genomes), we assume that our dataset represents a substantial fraction of the buckwheat genes. Among them are putative orthologs of genes that play key roles in the flower and inflorescence development, including those that are expressed at low level and are not widely represented in other transcriptome assemblies. These results are highly consistent with previous experimental and simulation-based studies [31-33] and provide further support for the use of combination of cDNA normalization and 454 sequencing for fast transcriptome characterization in non-model species. Application of the floral transcriptome sequence data for gene expression studies in *F. esculentum *mutants with altered flower development [34-36] will constitute the basis for the study of the genetic control of flower development in this species.

A fraction of contigs that did not produce any BLAST hits may also be an integral part of genomic data. The existence of such "non-blastable" sequences is reported in virtually all plant transcriptomes characterized to date, with their fraction varying from about 30 to 70% [23-25], depending on the species, the depth of sequencing and the parameters of BLAST search. There are two major groups of effects that may be responsible for this. The first group comprises the technical issues such as low quality of raw data, contamination by genomic DNA, inaccurate contig assembly and wrong choice of search parameters (e.g. too stringent e-value cut-off). The second group of effects, biological ones, is related to the characteristics of the data that are inherent to the species: the existence of non-coding cDNA fraction (primarily 5' and 3'UTR), lineage-specific genes (i.e. genes that are present in the genome being considered but absent from genomes represented in the databases) and fast-evolving genes, those that have orthologs in the other species but with a high level of sequence divergence precluding efficient recognition of orthologs.

In our study we tried to minimize the influence of technical effects. DNAse treatment of RNA was performed before cDNA synthesis to preclude genomic DNA contamination; also low quality data were excluded from the assembly process. As for the assembly accuracy, in the absence of a reference genome the possibility of assembly errors can not be completely ruled out. However given the relatively large length of 454-generated reads even chimeric combination of reads corresponding to different genes in one contig is unlikely to hamper efficient BLAST search. Indeed, assembly errors cause problems with similarity search only if short reads corresponding to small and weakly conserved fragments of different genes are combined into one contig.

Thus we assume that most of non-blastable contigs in our data are due to biological reasons. Some of them might indeed represent non-translated regions, but since the average length of predicted ORF in such contigs is significantly higher than in artificial sequences produced by random shuffling, we suggest that most of them correspond to protein-coding sequences, either lineage specific or highly variable. Currently there are no means to select one of these two options. Addition of more genomic and/or transcriptomic data for the Polygonaceae and other Caryophyllales is expected to improve the annotation and gene prediction in buckwheat.

The favourable effect of using the data from closely related species for annotation was reported for the analysis of chestnut (*Castanea*, Fagaceae) transcriptomes [32]. Several genes that could not be annotated using the information from the *Arabidopsis *proteome (*Castanea *and *Arabidopsis *belong to different clades of rosids - eurosids I and eurosids II) were successfully annotated when compared with *Populus *(belonging to eurosids I) proteome. This emphasizes that despite the availability of large-scale sequence data for model plant species and the increased rate of data acquisition for non-model species denser taxon sampling will further improve plant gene annotation. Given that the Caryophylalles is a group of exceptional interest from the point of view of evolutionary developmental biology we expect that more transcriptomic data will be available for this group.

### Comparison of two buckwheat transcriptomes: finding potential differentially expressed genes

Gene expression is often compared within one species at different developmental stages or in different conditions [22,37], and several efficient tools have been developed to analyze the enrichment in specific sets of genes (reviewed in [38]). The interspecific comparisons are rare and confined to the cases when it is possible to use sequence information from a phylogenetically close model species [39,40]. The possibility to use *de novo *sequencing data for the comparison of gene expression (even on the qualitative level, that is, to identify the presence or absence of expression) in different species has, to our knowledge, never been addressed in plant genomics.

Here we used an approach that is similar in its concept to the digital transcriptome subtraction [41]. The latter is based on the similarity search of sequences from one species against the other and selection of sequences that do not have significant matches. As expected, for most sequences from both datasets the sequences with high similarity were found in another species, either in contigs, or in singletons. However, about four thousands contigs in each species were found to be "species-specific". It is difficult to directly associate these contigs with the observed differences between *F. esculentum *and *F. tataricum*, the more so because about two thirds of them do not have significant BLAST hits in the nr database, and thus lack any functional annotation.

Still, some patterns are likely caused by species-specific expression. Firstly, the *F. esculentum *PDEG set shows a strong prevalence of contigs highly similar to retrotransposons. This suggests that either the *F. esculentum *genome has more retrotransposons than the genome of *F. tataricum*, or more of them are expressed. The former explanation seems to be more plausible, considering the fact that the genome size of *F. esculentum *is three-fold larger than that of *F. tataricum *[42]. While such differences in the genome size in plants are often caused by the polyploidy, it is not the case for the buckwheat since both species have the same chromosome number [42,43].

Secondly, the *F. esculentum *PDEG set is enriched in genes related to the disaccharide metabolism. Mono- and disaccharides, primarily glucose, fructose and sucrose, are a major component of the *F. esculentum *nectar [44]. *F. tataricum*, being a self-pollinated species, does not produce nectar. So the disaccharide metabolism genes indeed might be differentially expressed due to the difference in the nectar production. However, any conclusion about the differential expression should be treated with caution and verified by more precise methods such as quantitative RT-PCR. The set of "unique" contigs identified in our study is a mixture of genes whose orthologs are actually not expressed in the other species and those genes that are expressed in both species but are absent in the sequence data from one of the species due to some random fluctuations on different stages of RNA and cDNA processing. Indeed, our statistical analysis indicates that about 25-30% of PDEG are really differentially expressed. One of the possible issues responsible for this is the normalization because in normalized libraries more transcripts are represented and thus the probability of being sequenced is lower for each transcript than in case of non-normalized library.

The probability that a gene is expressed, given the observations of no reads from the other genome, is proportional to its coverage. It is very low for low-covered genes but among genes represented by a contig covered by more than 7 at least 95% of genes are expected to be differentially expressed (Table 5). Thus, further investigation of the genetic basis of specific differences between *F. esculentum *and *F. tataricum *may be conducted in two ways. The first one is the survey of the expression of PDEGs from the categories that are enriched in either species and that are likely to have species-specific expression for biological reasons (retrotransposons, sugar metabolism genes). The second one is the analysis of those genes that have a high number of reads in one transcriptome but are absent in thе other. A combination of these approaches may provide insights in the genetic and evolutionary mechanisms underlying morphological and physiological differentiation in the genus *Fagopyrum*. Further insights, including quantitative analysis of gene expression levels are also expected to result from the sequencing of non-normalized cDNA. This approach demonstrated its utility on several plant species [37,45].

### Phylogenetic utility of transcriptome sequence data

Besides its potential utility for functional genetic studies, the data from large-scale transcriptome sequencing is an important source of information for phylogenetic analysis. Phylogenetic studies based on transcriptome sequences yielded well-resolved and highly supported tree topologies for many groups of animals [46]. In plants, however, the phylogenomic approach has been mostly limited to plastid genome sequences [28]. There are two reasons for that. The first one is the relative scarcity of plant genome-scale data. Secondly, the starting point of any phylogenetic analysis, identification of orthologous genes, is a major problem for plants due to abundant genome-wide and small scale duplications.

Recently a set of single-copy genes was proposed for phylogenetic analysis on different taxonomic level. Though a number of articles reporting *de novo *plant transcriptome sequence data were published [25,33] none of them reported the use of these data for phylogenetic analysis. Here we attempted to validate the use of these genes for inferring angiosperm phylogeny by including in the analysis the data from four 454 transcriptome assemblies, with focus on Caryophyllales and asterids. Orthologs of most of these genes were found in all these transcriptomes. The tree inferred from the Bayesian analysis of these genes is similar to the ML tree from Duarte et al. [27] but has a higher resolution and node support. We suggest the increased resolution and node support reflects favourable effect of improved taxon sampling.

A major problem observed in our phylogenetic trees is the incongruence between different methods. The Bayesian inference and supertree (SDM) approach yield congruent topologies but these topologies are not congruent with those inferred from the maximum parsimony analysis. Also, MP analysis results in a tree with very low bootstrap support values. This may indicate that the MP is not applicable for transcriptomic data, where high variation is combined with lot of missing data. It is known that several types of data may require additional adjustments of procedures used for phylogenetic analysis (primarily alignment). For example, for non-coding sequences of plastid genomes special rules of alignment were developed and this increased resolution and reliability of phylogenetic trees inferred from these sequences [47,48]. The evolutionary peculiarities of plastid sequences are well studied [47,49]. On the contrary, little is known about the nuclear genes used for the phylogenetic analysis in this study. It is probable that the angiosperm-wide survey of the patterns of evolution of these genes will identify the source of incongruence between different methods of the analysis and, as the result, improve their utility for plant phylogenetics.

However, even under limited taxon sampling these genes provide valuable information on phylogenetic relationships within the angiosperms. The novel result inferred from these data is the strong support of sister relationships of Caryophyllales (including buckwheat) and asterids. Earlier we have shown that these relationships are strongly supported by the chloroplast genome sequence data [10]. It is well known however that the results of phylogenetic analysis of chloroplast gene datasets may be misleading and thus require verification by independent data [50,51]; the present study reports such verification for the phylogenetic position of Caryophyllales.

The results presented here emphasize that even shallow-coverage transcriptome sequence data are an important source of information for phylogenetic applications. The addition of such data from various lineages of flowering plants is expected to improve greatly the resolution, support and reliability of phylogenetic trees and to provide novel insights into the evolution of angiosperms.

## Conclusions

454 pyrosequencing of normalized cDNA libraries produced a large set of cDNA sequences for two congeneric plant species, *Fagopyrum esculentum *and *F. tataricum*. These sequences are an important resource for the evolutionary and developmental genetics in these species and in the order Caryophyllales. Analysis of single copy genes derived from transcriptome sequence data have great potential for inferring angiosperm phylogeny, especially with increased taxonomic sampling.

## Methods

### RNA extraction

Total RNA was extracted from developing inflorescences of *Fagopyrum esculentum *ssp. *ancestrale *and *F. tataricum *using the RNEasy Plant Mini kit (Qiagen) with following modifications: 50 μl of Plant RNA Isolation Aid reagent (Ambion) and 10 μl of b-mercaptoethanol were added to the lysis buffer RLT prior to homogenization and the homogenization was performed without liquid nitrogen. All other steps were performed according to the manufacturer's instructions. To avoid genomic DNA contamination RNA was treated with RNase-free DNase I (Qiagen). The RNA integrity was assessed by the agarose gel electrophoresis (1% gel with addition of SYBRGreen dye).

### cDNA synthesis, amplification and normalization and 454 sequencing

Total RNA was used for double-stranded cDNA synthesis using the SMART approach [52]. To increase the representation of weakly expressed genes, SMART-prepared amplified cDNA was then normalized using the DSN normalization method [53]. Normalization included cDNA denaturation/reassociation, treatment by a duplex-specific nuclease (DSN, [54]) and amplification of the normalized fraction. cDNA synthesis and normalization were performed by the Evrogen company, the detailed protocols are available in Additional file 6.

The efficiency of normalization was tested by real-time PCR. Fragments of *GAPDH *(a housekeeping gene expressed at a high level) and of the *WUS *ortholog (encodes a transcription factor expressed at a low level) were amplified from the normalized and non-normalized libraries. The 32-fold decrease of *GAPDH *expression was observed in the normalized library. Then 7 μg of normalized cDNA were fractionated and sequenced using 454 GS-FLX sequencer with 454 Titanium chemistry. Sequencing was performed by The Centre for Applied Genomics, The Hospital for Sick Children, Toronto, Canada. Each sample was run on a half of a picotiter plate.

### Assembly, annotation and comparative analysis of transcriptomes

SeqClean [55] was used to remove polyA sequences from the raw data. We did not use SeqClean to remove adapter sequences since it works only with 100-92% identity segments and in our case adapters contained many sequencing errors caused by the homopolymer runs. To remove the adapters with less than 92% identity we developed two scripts, which are available upon request. MIRA assembler [56] version 3.0.0rc4 was used for the 454 data assembly. The assembler was run in its 'accurate' mode with the assembly type set as 'EST'.

The resulting contigs were subject to the BLASTX search against the non-redundant protein database (nr) with the e-value threshold of 10^-6 ^and the HSP length cut-off of 50, as implemented in the BLAST2Go program [57]. The ORF Predictor tool was used to identify ORFs (http://proteomics.ysu.edu/tools/OrfPredictor.html, [58]). Searches against the *Arabidopsis *proteome (e-value threshold of 10^-6^) and the searches between *F. esculentum *and *F. tataricum *(e-value of 10^-15^) were performed using BLASTX/TBLASTN and BLASTN, respectively, as implemented in BioEdit 7.09 [59]. The Gene Ontology annotation [24] was done using BLAST2Go with the annotation cut-off of 10^-5^. We used simplified GOSlim (plant) annotations (developed by TAIR, [60]) to select genes expressed in the flower and in the inflorescence. For the identification of genes that are represented in the transcriptome of one species of buckwheat but are not represented in the other (here and further called potential differentially expressed genes, PDEG), a Perl script was developed that processes the BLAST output and extracts sequences that do not have significant matches. The enrichment analysis was performed using Fisher's exact test as implemented in Gossip/BLAST2Go [61]. The statistical assessment of the number of false positives in the PDEG sets was performed as follows. First, the probability *p(i,j,s) *to observe *i *reads for the gene in one species (*s) *given *j *reads for its ortholog in the other species was calculated. For each possible *j *and *s*, the observed number of orthologous pairs with *j *reads for gene in species *s *fitted well the Poisson model after filtering out a small number of outliers. The model parameters λ(*j*,*s*) were estimated from these data using the maximum-likelihood method, taking into account that pairs with zero number of reads for any of the genes were not observed for obvious reasons. Finally, the number of false positives was calculated as Σ *k*(*j*,*s*) p(0,*j*,*s*) where *k*(*j*,*s*) is the total number of genes with *j *reads in species *s*.

### Phylogenetic analysis

For the phylogenetic analysis, a set of 13 single copy genes from 69 taxa, from the [27] study was used. The orthologs of these genes were found by the BLAST search of corresponding *Arabidopsis *genes in three 454-sequenced transcriptomic datasets of Caryophylalles, *F. esculentum*, *F. tataricum*, and *Silene latifolia *(Filatov D.A., personal communication) and two transcriptomes of asterids: *Artemisia annua *[29] and *Craterostigma plantagineum *[22]. Since the orthologs from the two buckwheat species are highly similar (> 95%) the sequences from only one species (*F. tataricum*) used. Sequences of *F. tataricum*, *S. latifolia*, *A. annua *and *C. plantagineum *were added to the alignment from [27] then the whole set was checked for frameshifts and the latter, if present, were corrected by inserting N to recover in frame translation. Sequences of transcripts were translated into amino acid sequences by BioEdit and further aligned using MUSCLE ver. 3.6 [62]. The nucleotide sequence alignment was overlaid on the amino acid sequence alignment. Gap-rich positions and positions of ambiguous alignment were excluded from analyses (789 in total). The phylogenetic analysis using the maximum parsimony (MP) method was performed using PAUP* ver. 4.0b8 [63]. The MP analysis involved a heuristic search using TBR branch swapping and 500 random addition replicates. The non-parametric bootstrap analysis [64] was performed with 1000 replicates with TBR branch swapping. The Bayesian inference of phylogeny was explored using the MrBayes program ver. 3.1.2 [65]. The Bayesian analysis was applied both to the amino acid and nucleotide data with two runs with three chains in each. Each chain started with a random tree, 1,000,000 replicates for amino acid data and 5,000,000 replicates for nucleotide data were generated, trees were sampled every 100 generations. The number of discarded trees was determined using the cold-chain log-likelihood examination. The JTT model of amino acid substitutions was determined by the Bayesian information criterion in Modelgenerator ver. 0.43 [66], the GTR+I+Γ model of nucleotide substitution was determined by the Akaike information criterion in Modeltest ver. 3.7 [67], the data were treated as a single partition. As an alternative to the combined analysis a super distance matrix (SDM) method [68] was applied. The SDM-algorithm combines the evolutionary distances obtained from each gene into a single distance supermatrix, therefore for each gene a corresponding model of evolution was determined in Modeltest, then the maximum likelihood distances according to the selected model were computed with PAUP*. A supertree was constructed using Fitch program from PHYLIP package [69].

## List of abbreviations

AFLP: amplified fragment length polymorphism; BI: Bayesian inference; DSN: duplex-specific nuclease; GO: Gene Ontology; ML: maximum likelihood; MP: maximum parsimony; PCR: polymerase chain reaction; PDEG: potential differentially expressed genes; PP: posterior probability; RAPD: random amplified polymorphic DNA; RT-PCR: reverse transcription polymerase chain reaction; SDM: super distance matrix; SSR: simple sequence repeat

## Authors' contributions

MDL conceived and coordinated the study, performed transcriptome annotation and comparison and drafted the manuscript, ASK assembled contigs and developed scripts for the extraction of PDEG, DVV carried out statistical analysis of the PDEG, THS performed phylogenetic analysis, VJM and MSG participated in data analysis and interpretation and revised the manuscript, AAP participated in the design of the study and interpretation of the results and prepared RNA samples. All the authors read and approved the manuscript.

## Supplementary Material

Additional file 1 ***Fagopyrum esculentum *transcriptome sequence data**. The sequences of *F. esculentum *contigs and singletons and the summary of BLAST search and GO annotation of these sequences. Click here for file

Additional file 2 ***Fagopyrum tataricum *transcriptome sequence data**. The sequences of *F. tataricum *contigs and singletons and the summary of BLAST search and GO annotation of these sequences. Click here for file

Additional file 3 **Coverage of previously identified *Fagopyrum esculentum *genes**. Accession numbers of the sequences of *F. esculentum *genes known from previous studies and their coverage in the 454 transcriptome assembly. Click here for file

Additional file 4 **Distribution of GO categories in PDEG**. Distribution of GO categories for Biological Process (a) and Molecular Function subontologies (b) for *F. esculentum *(violet bars) and *F. tataricum *(blue bars) PDEG. Click here for file

Additional file 5 **SDM phylogenetic tree**. Phylogenetic tree inferred from the SDM analysis of 13 single-copy nuclear genes in 73 seed plant taxa. Scale bar corresponds to one substitution per ten sites. Click here for file

Additional file 6 **cDNA synthesis, amplification and normalization**. Detailed protocol of cDNA synthesis, amplification and normalization. Click here for file
