# Supplementary material for: De novo sequencing and characterization of floral transcriptome in two species of buckwheat (Fagopyrum)
Source: BMC Genomics. 2011 Jan 13;12:30. doi: 10.1186/1471-2164-12-30 (PMC3027159; doi:10.1186/1471-2164-12-30)
Supplement: Additional file 3 — Coverage of previously identified Fagopyrum esculentum genes. Accession numbers of the sequences of F. esculentum genes known from previous studies and their coverage in the 454 transcriptome assembly. [file 1471-2164-12-30-S3.DOC]

| accession number | putative function of gene product | reference | coverage |
| --- | --- | --- | --- |
| GU169469 | dihydroflavonol-4-reductase | Li et al. unpublished | 98.00% |
| AY826351 | aspartic proteinase 9 | Timotijevic et al. unpublished | 72.00% |
| GU172166 | chalcone synthase | Li et al. unpublished | 31.00% |
| D87984 | thioredoxin | Fujino et al. unpublished | 100.00% |
| GU363529 | phenylalanine ammonia-lyase | Li et al. unpublished | 57.00% |
| D87982 | legumin-like protein | Fujino et al. 2001 | 76.00% |
| D87980 | legumin-like protein | Fujino et al. 2001 | 69.00% |
| AF152003 | major allergenic storage protein | Nair et al. 1999 | 72.00% |
| EF488807 | BW10KD allergen protein | Jeon and Hong, unpublished | 100.00% |
| AB055892 | BW8KD allergen protein | Matsumoto et al. 2004 | 100.00% |
| AY256960 | 13S globulin | Samardzic et al. 2004 | 42.00% |
| FJ430161 | 16 kDa major allergen protein | Wang  et al. unpublished | 100.00% |
| DQ304682 | 16 kDa allergen protein | Koyano et al. 2006 | 100.00% |
| DQ681064 | metallothionein MT10 | Bratic et al. 2009 | 100.00% |
| DQ681063 | metallothionein MT5 | Bratic et al. 2009 | 100.00% |
| AF056203 | metallothionein-like protein | Maksimovic et al. unpublished | 100.00% |
| AB232935 | S-like RNase | Aii et al. unpublished | 94.00% |
| AB232934 | S-like RNase | Aii et al. unpublished | 92.00% |
| AB355645 | phytochelatin synthase | Mizuno et al. unpublished | 57.00% |
| DQ241824 | aspartic proteinase-like protein | Milisavljevic and Konstantinovic, unpublished | 29.00% |
| AY536047 | aspartic protease | Milisavljevic and Konstantinovic, unpublished | 29.00% |
| AB306326 | multidrug resistance-associated protein, | Mizuno et al. 2010 | 17.00% |
| DQ289792 | proteinase inhibitor BTIw1 | Li et al. 2006 | 100.00% |
| D87983 | protein declined during seed development | Fujino et al. unpublished | 43.00% |
| AF191099 | peroxiredoxin | Lewis et al. 2000 | 100.00% |
| AB290016 | autophagy-related protein 8 | Okabe et al. unpublished | 100.00% |
| AB290015 | hypothetical protein | Okabe et al. unpublished | 57.00% |
| AY126718 | fagopyritol synthase 1 | Ueda et al. 2005 | 19.00% |

**References**:

1. Fujino K, Funatsuki H, Inada M, ShimonoY, Kikuta Y. **Expression, cloning, and immunological analysis of buckwheat (*Fagopyrum esculentum* Moench) seed storage proteins.** *J. Agric. Food Chem.* 2001, 49 (4): 1825-1829.
2. Nair A, Ohmoto T, Woo SH, Adachi T. **A molecular-genetic approach for hypoallergenic buckwheat**. *Fagopyrum* 1999, 16: 29-36.
3. Matsumoto R, Fujino K, Nagata Y, Hashiguchi S, Ito Y, Aihara Y, Takahashi Y, Maeda K, Sugimura K. **Molecular characterization of a 10-kDa buckwheat molecule reactive to allergic patients' IgE**. *Allergy*, 2004, 59(5): 533-538.
4. Samardzic JT, Milisavljevic MD, Brkljacic JM, Konstantinovic MM, Maksimovic VR. **Characterization and evolutionary relationship of methionine-rich legumin-like protein from buckwheat**. *Plant Physiol. Biochem*. 2004, 42 (2): 157-163.
5. Koyano S, Takagi K, Teshima R, Sawada J. **Molecular cloning of cDNA, recombinant protein expression and characterization of a buckwheat 16-kDa major allergen**. *Int. Arch. Allergy Immunol* 2006, 140 (1): 73-81.
6. Bratic AM, Majic DB, Samardzic JT, Maksimovic VR. **Functional analysis of the buckwheat metallothionein promoter: tissue specificity pattern and up-regulation under complex stress stimuli**. *J. Plant Physiol.* 2009, 166 (9): 996-1000.
7. Li Y, Zhang Z, Liang A, Wang Z. **Cloning and characterization of a novel trypsin inhibitor (BTIomega1) gene from *Fagopyrum esculentum***. *DNA Seq*. 2006, 17 (3): 203-207.
8. Lewis ML, Miki K, Ueda T. ***FePer1*, a gene encoding an evolutionarily conserved 1-Cys peroxiredoxin in buckwheat (*Fagopyrum esculentum* Moench), is expressed in a seed-specific manner and induced during seed germination**. *Gene* 2000, 246 (1-2): 81-91.
9. Ueda T, Coseo MP, Harrel TJ, Obendorf RL. **A multifunctional galactinol synthase catalyzes the synthesis of fagopyritol A1 and fagopyritol B1 in buckwheat seed**. *Plant Sci.* 2005, 168 (3):681-690.
10. Mizuno T, Nakagawa M, Ono H, Sugiura D, Tamura H, Obata H. **Isolation of multidrug resistance associated protein like gene from lead hyperaccumulator common buckwheat and its lead detoxification ability**. *Plant Biotechnol*. 2010, 27: 39-46.

**Additional file 3. The coverage of *Fagopyrum esculentum* genes known from previous studies in the 454 transcriptome assembly.**
