# Supplementary figures and images for: De novo sequencing and characterization of floral transcriptome in two species of buckwheat (Fagopyrum)
Source: BMC Genomics. 2011 Jan 13;12:30. doi: 10.1186/1471-2164-12-30 (PMC3027159; doi:10.1186/1471-2164-12-30)

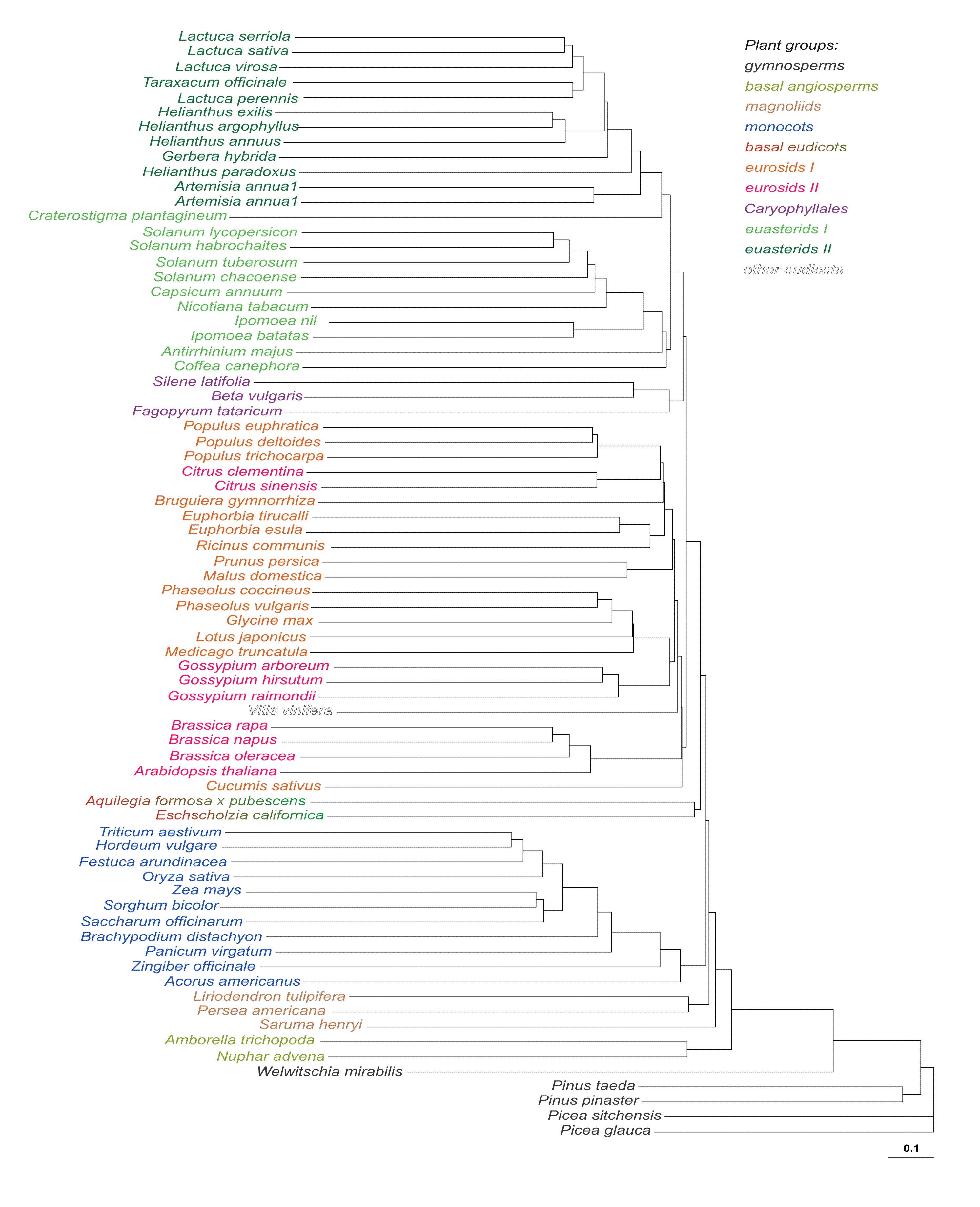

Supplement: Additional file 5 — SDM phylogenetic tree. Phylogenetic tree inferred from the SDM analysis of 13 single-copy nuclear genes in 73 seed plant taxa. Scale bar corresponds to one substitution per ten sites. [file 1471-2164-12-30-S5.JPEG]
