# Supplementary material for: De novo sequencing and characterization of floral transcriptome in two species of buckwheat (Fagopyrum)
Source: BMC Genomics. 2011 Jan 13;12:30. doi: 10.1186/1471-2164-12-30 (PMC3027159; doi:10.1186/1471-2164-12-30)
Supplement: Additional file 6 — cDNA synthesis, amplification and normalization. Detailed protocol of cDNA synthesis, amplification and normalization. [file 1471-2164-12-30-S6.DOC]

**Additional file 6. cDNA synthesis, amplification and normalization*.**

***First strand cDNA synthesis and cDNA amplification***.

Primer annealing mixture (5 µl) containing 0.3 µg of total RNA, 10 pmol of SMART Oligo II oligonucleotide (5’-AAGCAGTGGTATCAACGCAGAGTACGCrGrGrG-3’) and 10 pmol of CDS-T22 primer (5’-AAGCAGTGGTATCAACGCAGAGTTTTTGTTTTTTTCTTTTTTTTTTVN-3’) was composed and heated at 72°C for 2 min and cooled on ice for 2 min. Then it was complemented with the mix of Mint reverse transcriptase, 5X First-Strand Buffer, 2 mM DTT and 1 mM of each dNTP (up to a final volume of 10 µl). The first-strand cDNA synthesis reaction was incubated at 42°C for 2 hours in an air incubator and then cooled on ice. First-strand cDNA was then diluted 5 times with TE buffer, heated at 70°C for 7 min and used for amplification by polymerase chain reaction. PCR reaction (50 µl) contained 1 µl of diluted first-strand cDNA, 1 µl of 50x Encyclo PCR buffer, 200 µM of dNTPs, 0.3 µM of SMART PCR primer (5’-AAGCAGTGGTATCAACGCAGAGT-3’) and 1 µl of 50x Encyclo Polymerase mix. PCR was performed on MJ Research PTC-200 DNA Thermal Cycler under following program: 95°C - 7 seconds, 65°C - 20 seconds, 72°C - 3 minutes, 19 cycles for *Fagopyrum esculentum* and 18 cycles for *F. tataricum*. Amplified cDNA was purified using QIAquick PCR Purification Kit (Qiagen, USA) and concentrated by ethanol precipitation. DNA pellet was diluted by milliQ water to final cDNA concentration 50 ng/µl.

***cDNA normalization*.**

Normalization was performed using Trimmer cDNA Normalization Kit. First, hybridization was performed. Hybridization reaction contained 3 µl (about 150 ng) of purified ds cDNA and 1 µl of 4x Hybridization Buffer. The reaction mixture was overlaid with one drop of mineral oil and incubated at 98°C for 3 minutes and at 68°C for 5 hours. Then following preheated reagents were added to the hybridization reaction at 68°C: 3.5 µl of milliQ water, 1 µl of 5x DSN buffer and 0.5 µl of DSN enzyme. Then, incubation was prolonged at 67°C for 20 minutes. On completion of DSN treatment, DSN enzyme was inactivated by heating at 97°C for 5 minutes. Then cDNA sample was diluted by adding 30 µl of milliQ water and used for PCR amplification. PCR (18 cycles) was carried out using the same reagents and amplification program as described above.

*All reagents are from Evrogen, Russia if other is not indicated.
